# Cloning, reassembling and integration of the entire nikkomycin biosynthetic gene cluster into *Streptomyces ansochromogenes *lead to an improved nikkomycin production

**DOI:** 10.1186/1475-2859-9-6

**Published:** 2010-01-23

**Authors:** Guojian Liao, Jine Li, Lei Li, Haihua Yang, Yuqing Tian, Huarong Tan

**Affiliations:** 1 State Key Laboratory of Microbial Resources, Institute of Microbiology, Chinese Academy of Sciences, Beijing 100101, PR China; 2 Graduate School of Chinese Academy of Sciences, Beijing 100039, PR China

## Abstract

**Background:**

Nikkomycins are a group of peptidyl nucleoside antibiotics produced by *Streptomyces ansochromogenes*. They are competitive inhibitors of chitin synthase and show potent fungicidal, insecticidal, and acaricidal activities. Nikkomycin X and Z are the main components produced by *S. ansochromogenes*. Generation of a high-producing strain is crucial to scale up nikkomycins production for further clinical trials.

**Results:**

To increase the yields of nikkomycins, an additional copy of nikkomycin biosynthetic gene cluster (35 kb) was introduced into nikkomycin producing strain, *S. ansochromogenes *7100. The gene cluster was first reassembled into an integrative plasmid by Red/ET technology combining with classic cloning methods and then the resulting plasmid(pNIK)was introduced into *S. ansochromogenes *by conjugal transfer. Introduction of pNIK led to enhanced production of nikkomycins (880 mg L^-1^, 4 -fold nikkomycin X and 210 mg L^-1^, 1.8-fold nikkomycin Z) in the resulting exconjugants comparing with the parent strain (220 mg L^-1 ^nikkomycin X and 120 mg L^-1 ^nikkomycin Z). The exconjugants are genetically stable in the absence of antibiotic resistance selection pressure.

**Conclusion:**

A high nikkomycins producing strain (1100 mg L^-1 ^nikkomycins) was obtained by introduction of an extra nikkomycin biosynthetic gene cluster into the genome of *S. ansochromogenes*. The strategies presented here could be applicable to other bacteria to improve the yields of secondary metabolites.

## Introduction

Actinomycetes produce a wide variety of secondary metabolites, including antibacterial antibiotics, anticancer agents and immunosuppressive agents. Traditionally, the improvement of antibiotic-producing strain suitable for industrial fermentations is achieved by random mutagenesis and selection techniques. Although these techniques have succeeded in generating many industrial strains, it exhausted time and human resources. Overexpression of positive transcriptional regulators of a biosynthetic cluster or specific enzymes involving in metabolic bottlenecks has significantly overproduced a variety of natural products [1]. Whereas more than a single rate-limiting step often exists in the biosynthetic pathway of secondary metabolites [2], overexpression of the entire antibiotic biosynthetic gene cluster may possibly improve the yields of the desired natural product further.

The positive contribution of amplification of biosynthetic gene clusters to antibiotics production have been previously observed in some industrial overproducing strains [3-5]. For instance, in the kanamycin-overproducing strain, *Streptomyces kanamyceticus *12-6 which was generated by classical mutagenesis, tandem amplification of the entire kanamycin (Km) biosynthetic gene cluster was disclosed to directly contribute to Km overproduction and the level of Km production depended on the copy number of the Km biosynthetic gene cluster[5]. This provides a hint that introduction of extra copy of biosynthetic gene cluster into parent strain may be an effective and widespread approach to improve the production of antibiotics. However, the antibiotic biosynthetic gene cluster consisting of structural and regulatory genes is usually more than 30 kb, which is difficult to identify multiple unique restriction sties and circularize large vectors. So, few reports were related to overexpression of the whole biosynthetic gene cluster in parent strain using directed genetic approaches. Recently, the development of Red/ET technology enabled an alternative means of restriction/ligation-free gene manipulation of the large biosynthetic gene clusters. Red/ET technology is based on the discovery that in engineered *E. coli *containing Redα, Redβ, and Redγ proteins from the λ phage or RecE/RecT from the Rac phage, allelic exchanges can take place if a given DNA fragment is flanked at both ends by extensions of only a few tens of nucleotides that are homologous to a target DNA region [6,7]. Red/ET technology has become an efficient approach to cloning and modifying whole biosynthetic gene clusters. Biosynthetic gene clusters of myxochromide S (~30 kb) [8], myxothiazole (~60 kb) [9], coumermycin A1 (~38 kb) [10], phenalinolactone (~42 kb) [11], anthramycin (~32 kb) [12] and epothilone (~60 kb) [13] have been successfully constructed by different strategies based on Red/ET technology and heterologously expressed in different strains.

**Figure 1:**
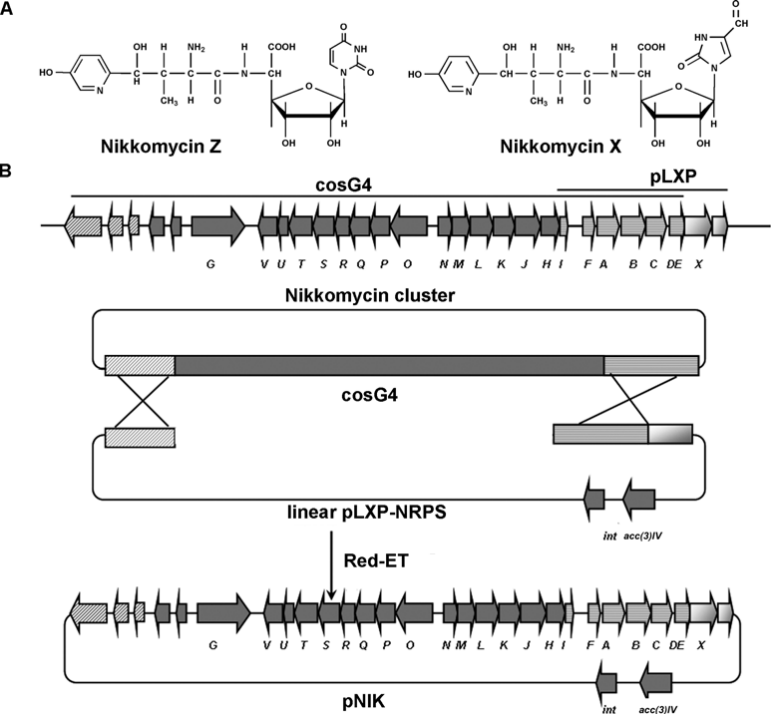
**Chemical structures of nikkomycin X and Z and strategy for reassembling the entire nikkomycin gene cluster in an integrative plasmid**. (A) Chemical structures of nikkomycin X and Z. (B) Construction of the nikkomycin biosynthetic gene cluster. The nikkomycin biosynthetic gene cluster consists of 22 ORFs (*sanG*-*sanX*) and is located on a supercos 1 based cosmid cosG4 and a pSET152 based plasmid pLXP. cosG4 contains most of the biosynthetic genes ranging from 8 kb DNA fragment upstream of *sanG *to partial *sanDE*; pLXP contains several biosynthetic genes ranging from partial *sanI *to 3 kb downstream of *sanX*. A 6 kb overlapping fragment between cosG4 and pLXP could be used as the lower recombinant arm. The upper arm was generated by insertion of a 4.5 kb DNA fragment from the upstream region of *sanG *into the pLXP to give pLXP-NRPS. The resulting pLXP-NRPS was linearized and then used to reassemble the whole cluster by Red/ET recombination with cosG4. The final construct containing the complete nikkomycin biosynthetic gene cluster was designated pNIK.

Nikkomycins are a group of peptidyl nucleoside antibiotics with potent fungicidal, insecticidal, and acaricidal activities. They are recognized as ideal fungicide and medications for human being by virtue of its nontoxic to mammals and bees, and easily degradable in nature [14]. Nikkomycin X and Z (Fig. 1A), the main components produced by *S. ansochromogenes*, consist of hydoxypyridylhomethreonine (nikkomycin D) and a 5-aminohexuronic acid *N*-glucosidically bound to uracil in nikkomycin Z or to 4-formyl-4-imidazolin-2-one (imidazolone) in nikkomycin X. The genes involving in nikkomycin biosynthesis have been identified and the coding regions responsible for the biosynthesis of nikkomycins spanned about 35 kb on the chromosome of *S. ansochromogenes *and organized in the three transcriptional units [15,16].

In this work, we report application of Red/ET technology combined with classic cloning procedures to construct the whole nikkomycin biosynthetic gene cluster in an integrative plasmid, and investigate the effect of duplication of the entire gene cluster on nikkomycins production in *S. ansochromogenes *and assess the stability of the engineered nikkomycin producer.

## Results

### Reassembling of the nikkomycin biosynthetic gene cluster by Red/ET

**Figure 2:**
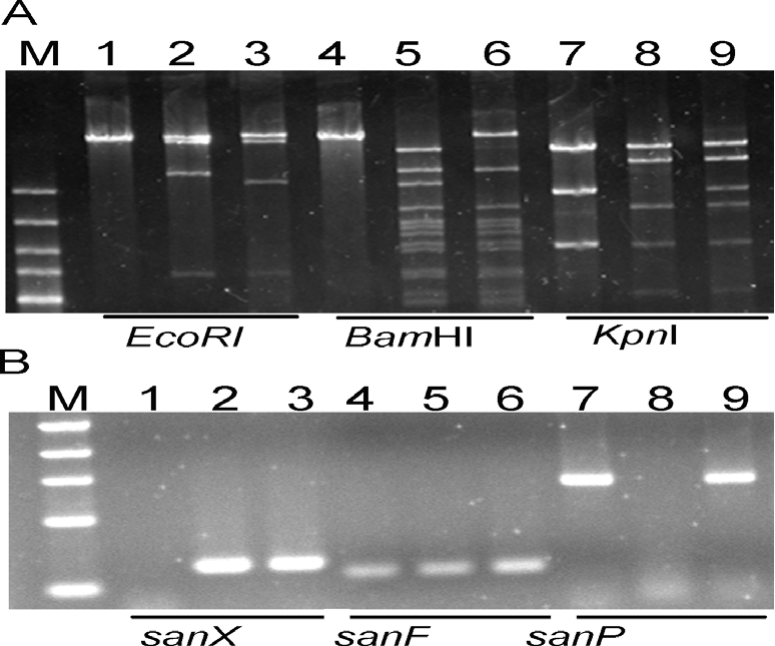
**PCR and restriction analysis of pNIK**. (A) Digestion of pLXP, cosG4 and pNIK with *Eco*RI, *Bam*HI and *Kpn*I. pNIK contained the same restriction fragments with pLXP and cosG4 and generated some new ones. M, 1 kb molecular marker; lane 1, 4 and 7, pLXP; lane 2, 5 and 8, cosG4; lane 3, 6 and 9, pNIK. (B) PCR analysis of pLXP, cosG4 and pNIK. pNIK contained all three genes tested. Primers sanP+, sanP-, sanX+, sanX-, sanF+ and sanF- were used to amplify a 400 bp, 220 bp and 120 bp DNA fragment of *sanP*, *sanX *and *sanF *using pLXP, cosG4 and pNIK as template. M, 100 bp molecular marker, lane 1, 4 and 7, cosG4; lane 2, 5 and 8, pLXP; lane 3, 6 and 9, pNIK.

Our previous studies showed that *sanG *and *sanX *were indispensable for nikkomycin biosynthesis and their disruption led to the abolishment of nikkomycin production [17,18], whereas deletion of a 8 kb upstream of *sanG *or 3 kb downstream of *sanX *did not affect nikkomycin production (data not shown). These results suggest that *sanG *is at the left boundary of the nikkomycin cluster and *sanX *is at the right boundary. To obtain the nikkomycin biosynthetic gene cluster, a cosmid library of genomic DNA from *S. ansocrhomogenes *7100 was constructed and screened by PCR. A cosmid named cosG4 containing most of the genes involving in nikkomycin biosynthesis was obtained. It harbored DNA sequences ranging from 8 kb upstream of *sanG *to *sanC *and partial *sanDE*, whereas intact *sanDE *and *sanX*, which were also essential for nikkomycin biosynthesis, were not located on cosG4 (Fig. 1B). pLXP, a plasmid based on pSET152, had an 11 kb insertional sequence containing the partial *sanI *and complete *sanF, sanA, sanB, sanC, sanDE, sanX *and 3 kb downstream of *sanX *[18]. cosG4 and pLXP harbored 6 kb overlapping regions, which enabled the use of Red/ET recombination to reassemble both parts to construct the nikkomycin biosynthetic gene cluster (NIK gene cluster). Reconstruction of the NIK gene cluster in a single plasmid was performed as described in the section of Materials and Methods(Fig. 1B)and the final construct was designated as pNIK. The identity of pNIK was further confirmed through PCR and restrictive analysis (Fig. 2).

### Duplication of the NIK gene cluster in *S. ansochromogenes *7100

pNIK was passed through *E. coli *ET12567/pUZ8002 and then introduced into *S. ansochromogenes *7100 by conjugal transfer according to established techniques. The resulting transformants were inoculated on MS plates in the presence of apramycin to form spores. Gray spores were harvested and transferred into liquid medium.

**Figure 3:**
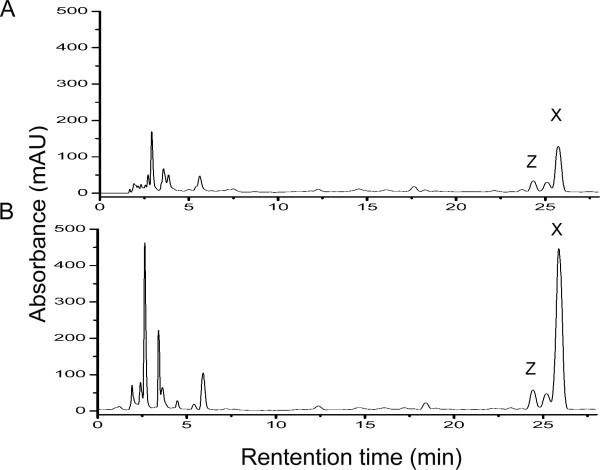
**HPLC analysis of Nikkomycins production in *S. ansochromogenes *and its derivatives**. (A) *S. ansochromogenes *7100 (pSET152); (B) DNik. The strains were inoculated in liquid SP medium for 120 h. X, nikkomycin X; Z, nikkomycin Z.

**Figure 4:**
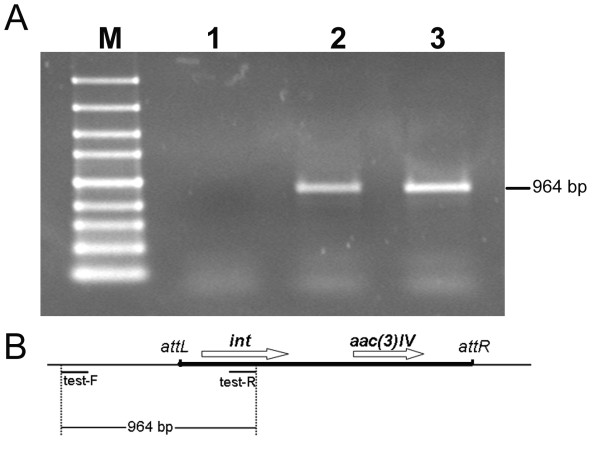
**PCR analysis of the integration of pNIK into the genome of *S. ansochromogenes***. (A) M, 100 bp marker; Lane 1, *S. ansochromogenes *7100; Lane 2, *S. ansochromogenes *7100 (pSET152); Lane 3, *S. ansochromogenes *DNik. (B) Physical map of pSET152 inserted into the *attB *site of *S. ansochromogenes*. Primer test-F and test-R were used to amplify 964 bp DNA fragment from the genomic DNA of *S. ansochromogenes *7100 (pSET152) and DNik. Chromosomal DNA is indicated by thin lines, and pSET152 is indicated by thick lines. *int*, the integrase gene; *aac(3)IV*, the apramycin resistance gene.

**Figure 5:**
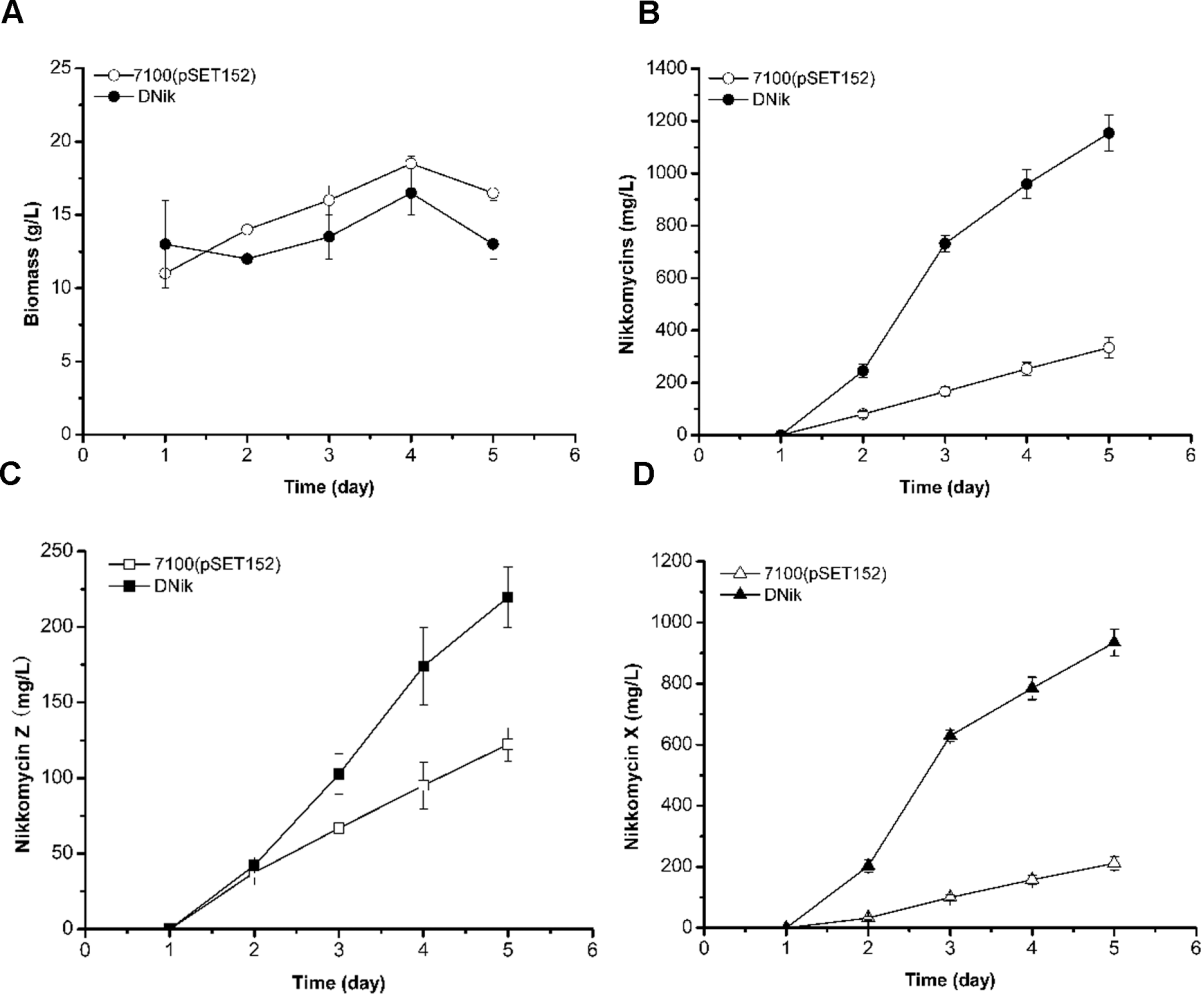
**Nikkomycins production and biomass of *S. ansochromogenes *and its derivatives in SP medium**. (A) Biomass. *S. ansochromogenes *7100 (pSET152) (open circle), DNik (close circle); (B) Total nikkomycins production; (C) Nikkomycin Z production. *S. ansochromogenes *7100(pSET152) (open square), DNik (close square); (D) Nikkomycin X production. *S. ansochromogenes *7100(pSET152) (open triangle), DNik (close triangle).

Three apramycin-resistant transformants were obtained and all of them led to an improved nikkomycin production ranging from 4-fold to 5-fold (Fig. 3). As control, introduction of pSET152 into the chromosome of *S. ansochromogenes *had no effect on nikkomycin production (data not shown). One of the tranformants, designated DNik, was chosen for further investigation. To verify the integration of pNIK into the genome of *S. ansochromogenes*, genomic DNA of *S. ansochromogenes *7100, *S. ansochromogenes *7100 (pSET152) and DNik was isolated and used as template to perform PCR. A 964 bp of DNA fragment was amplified from genomic DNA of *S. ansochromogenes *7100 (pSET152) and DNik, but not from *S. ansochromogenes *7100 (Fig. 4), demonstrating that pNIK integrated at the *attB *site in the genome of *S. ansochromogenes*. The *S. ansochromogenes *7100 (pSET152) and DNik had comparable growth rates and final biomass (Fig. 5A), indicating that higher biomass was not an overproduction mechanism of DNik. DNik showed higher nikkomycins production than *S. ansochromogenes *7100 (pSET152) after incubation for 2 days, and 4.3-fold (1100 mg/L) much higher production after 5 days (Fig. 5B). Furthermore, introduction of pNIK into *S. ansochromogenes *7100 had different effect on the yields of nikkomycin X and nikkomycin Z, leading to 4-fold (880 mg/L) improvement of nikkomycin X production and 1.8-fold (220 mg/L) of nikkomycin Z (Fig. 5C and 5D).

To investigate the stability of DNik, the mutant was incubated in the absence of apramycin. After 5 passages, 10 colonies were randomly selected and cultured in the presence of apramycin for 5 days. All of them still conferred apramycin resistance and produced approximately 4.2-fold of yields of nikkomycins compared with the parent strain (data not shown). These results demonstrated that the transformants harboring one extra copy of NIK gene cluster are genetically stable in *S. ansochromogenes*.

## Discussion

Duplication of biosynthetic gene clusters to increase the yields of secondary metabolites had been applied using directed genetic approaches. In fungi, introduction of the whole biosynthetic gene cluster of penicillin into penicillin producing strain *Penicillium chysogenum *Wis54-1255 and that of compactin into *P. citrinum *resulted in significantly increased production of penicillin and compactin, respectively [19,20]. In *Streptomyces*, when the whole gene cluster of cephamycin C from *S. cattleya *was introduced into another cephamycin producer, *S. lactamgens*, a 2 to 3-fold improvement in titer was achieved [21]. However, in these cases, cosmids were applied to load the antibiotic gene clusters. Isolation of an intact biosynthetic gene cluster onto a single comsid was extremely difficult owing to its large size. Instead, construction of the whole biosynthetic gene cluster from several cosmids or plasmids which contained partial DNA fragments of the cluster was more feasible and the development of Red/ET technology facilitated this procedure. In this work, we employed Red/ET technology to reassemble the entire NIK cluster in an integrative vector, pSET152, which potentially allowed the resulting nikkomycin overproducer with genetic stability and demonstrated positive effect of introduction of NIK cluster into the parent strain on nikkomycins production. Our results were consistent with other groups' similar results, showing that duplication of NIK cluster could significantly improve the yields of nikkomycins.

It is common that high-producing strains containing a self-replicating plasmid or cosmid usually lose their high producing ability when they are incubated in the absence of antibiotic resistance selection pressure [21]. However, it was impractical to culture strains in large scale in the presence of antibiotics. pSET152, which could be introduced into *Streptomyces *by conjugal transfer and stably integrated into the *Streptomyces *chromosome by site-specific recombination at the phage φC31 attachment site (*attB*) [22], provided an alternative to get stable high-producing strains. It was feasible to construct the whole secondary biosynthetic gene cluster using Red/ET technology into pSET152 or other vectors which contained the DNA fragments of *oriT *(RK2) region, the phage φC31 attachment site and integrase gene from pSET152. As a linear relationship was observed between the cluster copy number and the yields of antibiotics [3,5], more copies of the gene cluster could be introduced into the *Streptomyces *chromosome using other phage based plasmids (e.g. pSAM2, pSOK804 or pRT801), which integrated at sites different from the φC31 [23]. Recently, Tyo and colleagues developed an efficient approach to increase the copy number of target DNA fragment in *E. coli *using chemically inducible chromosomal evolution (CICHE) [24] and its application in *Streptomyces *would facilitate the introduction of more copy of whole biosynthetic gene cluster into the *Streptomyces *chromosome. It is noteworthy that the highest nikkomycin production reported is 3.2 g/L in *S. tandae *Tü 901/S 2556, which was subjected to multiple rounds of strain improvement [25]. Therefore, we expect that introduction of extra copy of NIK cluster could be a potential strategy for further increase of nikkomycin production.

Duplication of the whole NIK cluster had different effect on the nikkomycin X and nikkomycin Z production. Nikkomycin X and Z had the same peptidyl moiety but different nucleoside moiety. Imidazolone, the base of nikkomycin X, was catalyzed by enzymes encoded by *sanO, sanQ *and *sanP *from *S. ansochromogenes *[26], whereas uracil, the base of nikkomycin Z, was synthesized by *de novo *synthesis or salvage pathway. Duplication of the NIK gene cluster may increase the transcription of the whole transcriptional units, providing more precursors for biosynthesis of nikkomycin X. However, duplication of the NIK gene cluster could not significantly increase the pool of uracil, resulting in a modest increase in nikkomycin Z production (1.8-fold). These results were consistent with our previous results that overexpression of *sanO *could only increase the yields of nikkomycin X [27].

## Conclusion

Development of new techniques and tools for gene manipulation (e.g. Red/ET recombination) significantly facilitate cloning and reassembling of antibiotic gene clusters with large size in a given vector. In this study, Red/ET approach was applied to reassemble the entire nikkomycin biosynthetic gene cluster in an integrative vector, allowing the generation of a stable nikkomycin overproducer. Our investigation provides an insight that introduction of the biosynthetic gene cluster into parent strain could be a simple and widespread approach for improving the yields of antibiotics of commercial interest.

## Materials and methods

### Bacterial strains, plasmids and growth conditions

Bacterial strains and plasmids used in this study are listed in Table 1. *S. ansochromogenes *7100, the wild type producer of nikkomycin (120 mg/L nikkomycin Z and 220 mg/L nikkomycin X), was collected in our laboratory. *S. ansochromogenes *and its derivative strains were grown at 28°C in different media. Liquid medium YEME and solid medium MS were prepared as described elsewhere[28]. SP medium (3% mannitol, 1% soluble starch, 0.5% soy peptone and 0.8% yeast extract, pH 6.0) was used for nikkomycins production. *E. coli *strains were incubated in LB medium at 37°C or 30°C (for Red/ET protein expression). When necessary, antibiotics were used at the following concentrations: apramycin, 10 μg ml^-1 ^in YEME or MS for *S. ansochromogenes*, 100 μg ml^-1 ^in LB for *E. coli*; kanamycin, 10 μg ml^-1 ^in YEME or MS for *S. ansochromogenes*, 100 μg ml^-1 ^in LB for *E. coli *and choramphenicol 34 μg ml^-1 ^in LB for *E. coli.*

**Table 1:** Strains and plasmids used in this study.

Strains or plasmids	Relevant characteristics	Sources
Bacterial strains		
*S. ansochromogenes*		
7100	Wild-type strain	[31]
DNik	*S. ansochromogenes *7100 containing plasmid pNIK	This work
*E. coli*		
DH5α	F-*recA*, f80, d*lacZ *ΔM15	Gibco BRL
ET12567 (pUZ8002)	*recE, dam, dcm, hsdS*, Cm, Str, Tet, Km	[32]
XL 1-blue MR	Δ*(mcrA)183*,Δ*(mcrCB-hsdSMR-mrr)173, endA1, supE44, thi-1, recA1, gyrA96, relA1, lac*	Stratagene
BW25113	K12 derivative: Δ*araBAD*, Δ*rhaBAD*	[33]
Plasmids		
pBluescript KS-	Routine cloning and subcloning vector	Stratagene
pBS	Digestion of pBluescript II KS- with *BamH*I-*Pst*I, blunt-ended and religation.	This work
supercos 1	Vector for cosmid library construction	Stratagene
pSET152	Integrative vector	[22]
pIJ790	λRED (*gam, bet, exo*), *cat, araC, rep101*^*ts*^	[33]
cosG4	A cosmid containing most of the nikkomycin biosynthetic genes	This work
pLXP	11 kb DNA fragment containing partial nikkomycin biosynthetic genes inserted into pSET152	[18]
pNIK	A pSET152 based plasmid containing the whole nikkomycin biosynthetic gene cluster	This work

### Primers and Polymerase chain reactions

Polymerase chain reactions (PCRs) were performed using Taq DNA polymerase. sanP+: GCGGCCAGCTACTTCCGGGAC, sanP-: GCAGAAAGGCCGAGCGCATGT; sanX+: GCAGAAAGGCCGAGCGCATGT, sanX-: GCTACAGCGGGGCGGTCAAG; sanF+: CAGGTCGGGGATCAGGGTC, sanF-: GCGGCCAGCTACTTCCGGGAC; all of them were used to confirm the identity of pNIK. An initial denaturation at 95°C for 5 min was followed by 30 cycles of amplification (95°C for 1 min, 60°C for 30 s and 72°C for 1 min) and additional 10 min at 72°C.

### Construction of genomic library for *S. ansochromogenes*

For construction of a cosmid library of *S. ansocrhomogenes*, 10 μg of chromosomal DNA was partially digested with *Sau*3AI. The 30-45 kb DNA fragments were isolated and ligated into supercos 1. After incubation at 16°C for 16 h, the ligated DNA was packaged using Gigapack III XL packaging kit (Stratagene) and introduced into *E. coli *XL-blue MR. The cosmid library was screened by PCR using primers sanP+ 5' GCGGCCAGCTACTTCCGGGAC 3' and sanP- 5' GCAGAAAGGCCGAGCGCATGT 3'. Cosmid (mix) was used as template and the positive clones were identified in 96-well plates.

### Reassembling of the entire nikkomycin biosynthetic gene cluster by Red/ET

The Red/ET recombination technique was described previously [6]. In the present study this method was used for reassembling the whole nikkomycin biosynthetic gene cluster onto an integrative plasmid.

A 4.5 kb *EcoR*I-*Xho*I DNA fragment containing the upstream region of *sanG *from cosG4 was inserted into the same site of pBS-1 to generate pNRPS, and then digested with *Xba*I/*Bam*HI. The resulting 4.5 kb DNA fragment was inserted into the same site of pLXP to give pLXP-NRPS. Subsequently, pLXP-NRPS was digested with *Bam*HI, dephosphorated with calf phosphatase (Takara, Japan) to generate the two recombinant arms for Red/ET recombination. The resulting linear fragment (1 μg) was then introduced into *E. coli *BW25113/pIJ790 containing cosmid cosG4 by electroporation. The pNIK was selected by apramycin resistance.

### Integration of pNIK into the genome of *S. ansochromogenes*

phiC31 integrase in pSET152 can integrate intragenically into the *attB *site of *SCO3798 *of *S. coelicolor *[29]. *SCO3798 *is a highly conserved gene in *Streptomyces *and its homologue (92% identity) is found in genome of *S. ansochromogenes *after whole genome 454 sequencing (Li, *et al*, unpublished results). Primer test-F: GGTGTCGCCGTTGGTGATG and test-R: GGCTTGAAGGGAAGGTGTTTGT were used to verify the integration of pSET152 or pNIK into the genome of *S. ansochromogenes*.

### HPLC analysis of nikkomycins

Spores of *S. anchromogenes *were inoculated in YEME. The cultures were grown at 28°C on a rotary shaker (220 rpm) for 48 h and used as seed cultures. 1 ml (0.5% V/V) of seed culture was inoculated into flasks containing 50 ml of SP medium, and then fermented at 28°C on a rotary shaker (200 rpm) for 5 days. The culture filtrates were harvested by centrifugation and the supernatant was filtered through a Minipore membrane (pore diameter 0.2 μm). Nikkomycins were identified by HPLC analysis (Agilent 1100 HPLC and RPC-18) at 290 nm absorption wavelengths. Chemical reagent, mobile phase and gradient elution process were as described by Fiedler [30].

## Competing interests

The authors declare that they have no competing interests.

## Authors' contributions

GL and JL carried out the experiments and analyzed the primary data. GL wrote the draft manuscript. LL, HY and YT assisted with experimental design and data analysis. HT supervised the whole work and revised the manuscript. All authors read and approved the final manuscript.
